# Environmental Enrichment as Part of the Improvement of the Welfare of Japanese Quails

**DOI:** 10.3390/ani12151963

**Published:** 2022-08-02

**Authors:** Anastasiya Ramankevich, Karolina Wengerska, Kinga Rokicka, Kamil Drabik, Kornel Kasperek, Agnieszka Ziemiańska, Justyna Batkowska

**Affiliations:** 1 Student Research Group of Poultry Biology, Breeding and Management, University of Life Sciences, 13 Akademicka St., 20-950 Lublin, Poland; ramankevichanastasiya@gmail.com (A.R.); karolina.wengerska@up.lublin.pl (K.W.); rokicka.kingaa@gmail.com (K.R.); 2 Institute of Biological Basis of Animal Production, University of Life Sciences, 13 Akademicka St., 20-950 Lublin, Poland; kornel.kasperek@up.lublin.pl (K.K.); agnieszka.ziemianska@up.lublin.pl (A.Z.); justyna.batkowska@up.lublin.pl (J.B.)

**Keywords:** behaviour, cortisol, corticosterone, behavioural tests

## Abstract

**Simple Summary:**

Nowadays, keeping animals in cage systems is controversial. Regulations and increased consumer awareness are causing cage systems to lose ground in many countries. In the case of Japanese quails bred for both egg and meat production, their maintenance in a non-cage system is very difficult and uneconomical; therefore, they are kept in cages, and, unlike egg-laying hens, the regulations only regulate the basic parameters, such as cage size and access to food and water. Taking this into account, studies have been conducted that indicate that keeping quails in enriched cages improves the birds’ welfare.

**Abstract:**

The aim of this study was to evaluate the indicators of the behavioural and physiological welfare of Japanese quails (*Coturnix japonica*) as possible responses to the enrichment of the birds’ habitat. The study sample consisted of 280 Japanese quails (224 ♀ and 56 ♂, respectively). Birds of 5 weeks of age were randomly divided into seven equally sized groups and then divided into replication subgroups (four per group, 10 birds in each replication). Birds were maintained in 0.5 m^2^ cages with unrestricted access to water and food. The experimental factor was the presence or absence of enrichment of the birds’ cages: the nest box, scratcher, plastic corrugated pipe (tunnel), limestone cubes, sandbathing box and feeder box with a drilled cover. Quails were subjected to behavioural tests (tonic immobility and open field tests) and, after 6 weeks, blood samples were taken from them to determine their biochemical indices as well as their cortisol and corticosterone levels. An additional element was the assessment of fertility indices. The presence of enrichment was shown to reduce behavioural disturbances in Japanese quails. This study also found that the colour and shape of an object were very important regarding the birds’ interest in it. Additionally, individuals kept in enriched cages, who were allowed to exhibit their natural behavioural patterns, had lower stress levels.

## 1. Introduction

Consumer interest in the raw materials obtained from birds kept in welfare states has been increasing recently. This is forcing meat and egg producers to make changes to their flock management in order to meet the requirements of consumers of poultry raw materials [1]. In the case of certain species or systems, appropriate regulations have already been implemented to preserve the animals’ welfare in commercial farming. A flagship example in this field is egg-laying hens, especially those kept in cages, for which the Council Directive 1999/74/EC [2] has been issued, regulating the necessary equipment in the cages.

The concepts of welfare are numerous and cannot be formulated unambiguously. According to Broom [3], welfare is the state in which the animal has to “ manage” (or “deal with”) factors in its environment. However, from the definition given by Hughes [4], it can be concluded that welfare is a state of physical and mental health, where the animal is in complete harmony with its environment, equivalent to the ability of the animal to maintain homeostasis in its body. Providing the animal with appropriate living conditions allows it to feel comfortable both physically and mentally.

In the context of bird welfare, research hitherto conducted has identified several factors that can disrupt bird welfare to varying degrees. These occur at all stages of rearing, from the time of hatching [5] through to beak-correction treatments [6] and treatment associated with moving the birds between production buildings, or concerning changes to their housing systems [7].

Despite the complexity of the concept of welfare, it is measurable indirectly through the analysis of physiological indices (plumage, fertility rate, heterophil/lymphocyte ratio and technopathies), biochemical parameters, production effects (laying rate, weight gain, loss of body weight, feed intake and conversion ratio) and behaviour (the occurrence of feather pecking, stereotypy and the duration of tonic immobility). Behavioural traits are among the foremost reliable and observable indicators of poultry welfare. The inability of animals to manifest their natural behavioural patterns causes them frustration, which in turn is manifested through the occurrence of stereotypies or behavioural abnormalities. Low [8] indicates that stereotypies in poultry are characterised by excessive vocalisation, locomotion, walking in circles and pecking at cage bars. Some of the behaviours, such as frequent feather combing and feather shaking, allow birds to relieve stress [9]. Among the behavioural disorders associated with bird welfare disorders is feather pecking, which, despite its comprehensive aetiology, is most frequently associated with bird welfare disorders [10,11].

Although birds’ welfare has recently become a “top” topic in many scientific papers and public discussions, it is almost exclusively reserved to laying hens and broiler chickens. Other species, probably due to their lower popularity, are somewhat disregarded.

For example, Japanese quails (*Coturnix japonica*) are becoming increasingly popular in the contexts of both table eggs and meat production. For the first purpose, females begin laying at about 40 days of age [12], with a productivity rate of over 250 eggs during the production cycle [13,14]. For the purpose of reproduction, Japanese quails are kept in flocks consisting of one male and four females [14]. At the same time, it should be noted that, unlike their wild ancestors, these birds do not have brooding instincts [15].

Japanese quails (*Coturnix japonica*), due to their small body size, low feed intake and short generation interval, are used as model animals in many studies [16] and are typically reared in a cage system. However, no minimum housing standards (except for basic stocking density recommendations) have been defined for quails, as have been defined for caged laying hens.

According to the legislation adopted for quail cages, producers are only obliged to provide the birds with feeders and drinkers. Therefore, all additional elements introduced into the birds’ habitat can be considered as enrichments. Very few reports deal with the potential enrichment of the Japanese quails’ environment. Scratchers [17], bottle caps, coloured wool and wooden platforms [18], or sand-bath devices and ropes or synthetic grasses [19], have been used for this purpose. However, the aforementioned studies aimed to investigate the effects of introducing bird cage enrichments on the induced stress responses. In contrast, there is a lack of data on the effects of enrichment on the typical rearing and breeding stress responses of Japanese quails; therefore, it seems to be necessary to place the emphasis on their welfare. The aim of this study was to assess the possibility of improving the welfare of Japanese quails (*Coturnix japonica*) by enriching their habitat.

## 2. Materials and Methods

Approval from the local ethical committee for animal experiments (No. 17/2021) was obtained before the start of this research.

The study material consisted of 280 Japanese quails, including 224 females (♀) and 56 males (♂), individually marked with wing marks. Birds of 5 weeks of age were sexed and randomly divided into 7 equally sized groups, each with 4 replication subgroups (10 birds in each replication, with a 1♂: 4♀ ratio). The experiment started after 7 days, during which the birds were acclimatised to the new environment and to the presence of the staff. Birds were maintained in 0.5 m^2^ mesh cages with a grid floor (mesh size 0.5 × 0.5 cm). The cages were placed on the same level and arranged side-by-side along the long walls of the building. A standard photoperiod (17 h of light:7 h of darkness), continuous ventilation and optimal temperature (18 °C) were maintained, and the birds had unlimited access to water and food.

**Figure 1:**
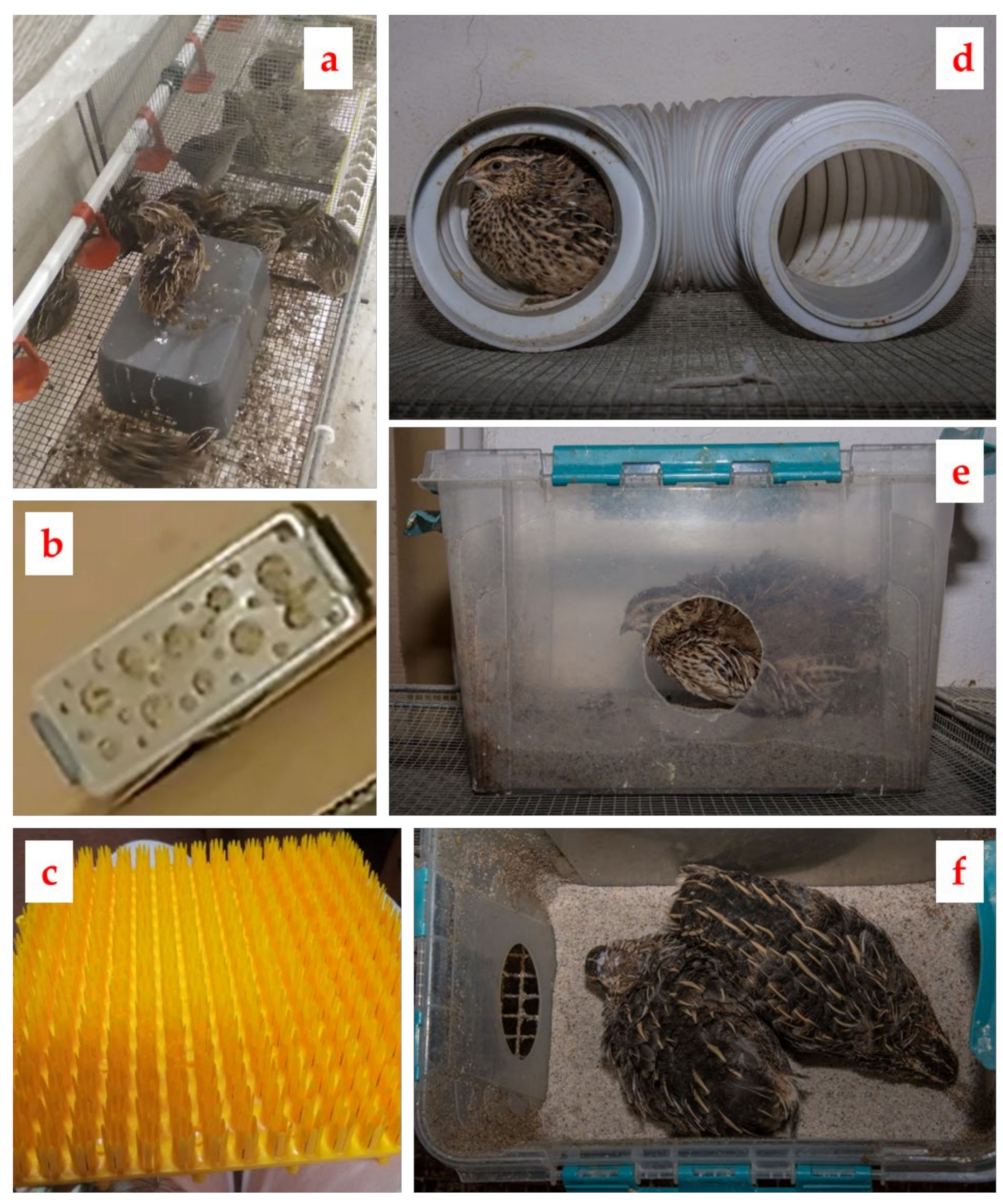
The enrichments applied ito the Japanese quail cages ((**a**)—nest box, (**b**)—feeder, (**c**)—scratching surface, (**d**)—tunnel, (**e**,**f**)—sand box).

The experimental factor was the presence or absence of enrichment of the cages. Group 1 (the control) was kept in typical cages, equipped only with feeders and nipple drinkers. In the cages of group 2 (the nest), the nest box—a plastic box with non-transparent walls and no bottom (25 × 25 × 15 cm) with 2 cut-outs—was placed on the floor. In the cages of group 3, the birds had a plastic pine needle mat as a scratching area (15 × 15 cm) attached to the floor of the cage. In the cages of group 4 (the tunnel), the flexible corrugated plastic pipe was laid on the bottom of the cage (60 cm long and approx. 15 cm in diameter). In each cage of the 5th group (the limestone cube), 2 limestone cubes were placed on the wall 7 cm above the bottom of the structure. The cages of group 6 (the sand) were equipped with transparent plastic containers (25 × 25 × 25 cm) with 2 circular cut-outs (at 5 cm from the bottom of the structure, diameter 7 cm). The box was filled with loose material (autoclaved sand). The last group, the 7th (the feeder), had cages equipped with a flat, non-transparent plastic container (15 × 21 × 3 cm) with a drilled cover, and the diameter of holes varied (0.2, 0.3, 0.5, and 0.7 cm). This box was filled with the food mixture. A more detailed experimental scheme is shown in Table S1. In Figure 1, pictures of some of the enrichments are shown.

As one of the elements related to the quails’ behaviour, the daily recording of the egg-laying site was carried out throughout the experimental period. Additionally, in week 6 of the experiment, 100 eggs from each group were collected to verify the effects of the applied experimental factors on the fertility rate. The eggs were incubated (up to day 6) under the standard conditions (specific for Japanese quails), using an automatic hatching apparatus manufactured by Jarson^®^ (Gostyń, Poland). The proportions of fertilised to laid set eggs were evaluated.

**Figure 2:**
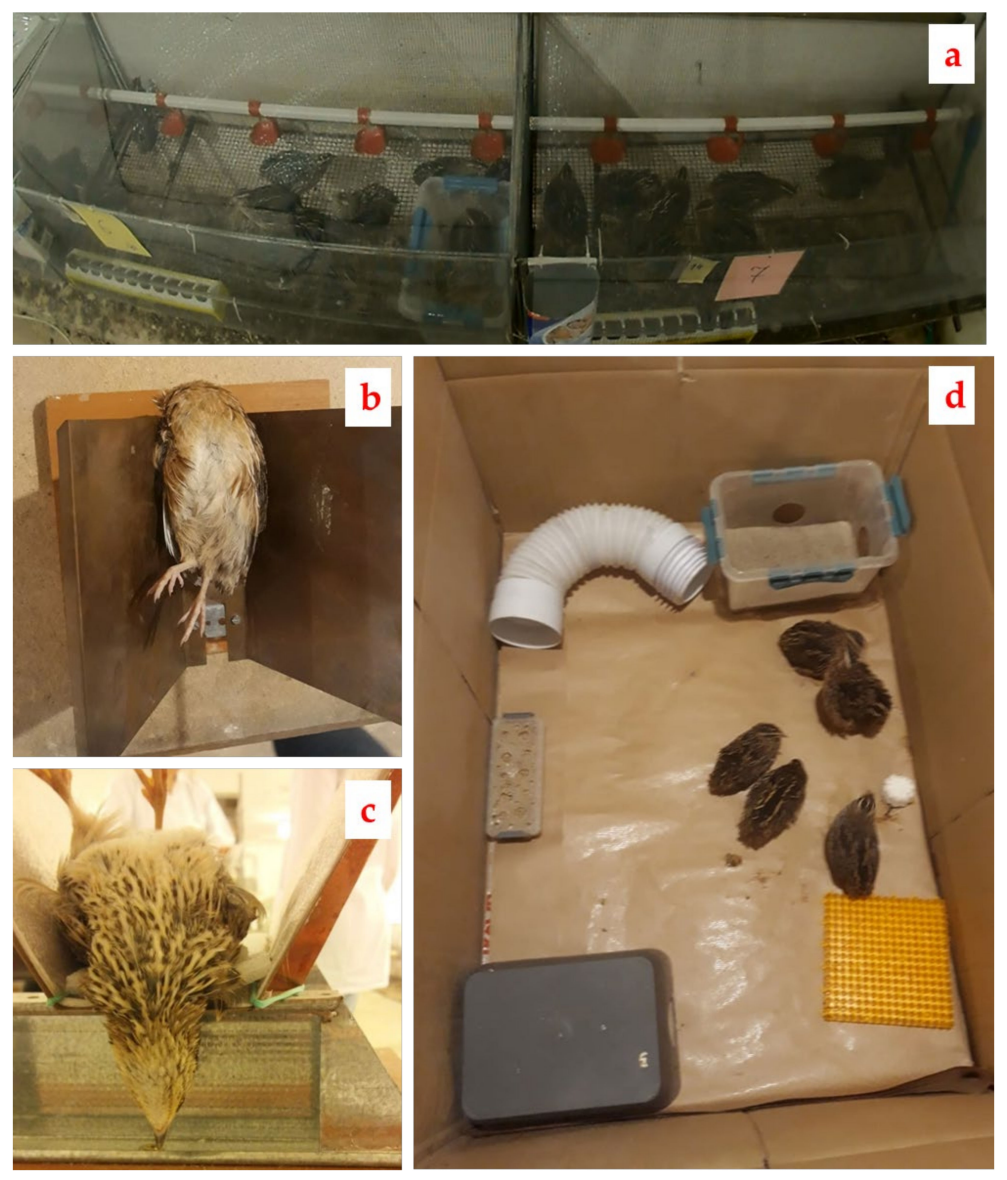
The behavioural tests performed during the experiment ((**a**)—cage observation, (**b**,**c**)—tonic immobility test, (**d**)—enriched open-field test).

In terms of the welfare and behavioural changes under the influences of the elements introduced to the quails’ environment, 2 behavioural tests were carried out (Figure 2):

- An enriched open-field test, in which an arena with dimensions of 100 × 100 × 80 cm (length × width × height) was enriched with all the objects placed previously in the cages. Each enrichment was placed at separate locations in the arena, and the arena floor was divided into squares in order to more easily determine the quails’ paths of movement. A total of 5 birds from each group were randomly selected (1♂:4♀) and placed in the centre of the arena. The test was carried out over 15 min for later analysis. The choice of the object, the distance covered by the individuals to reach the object, and the time the animals spent with the object were evaluated. The object choice was scored on a 0–1 scale, where 1 was given if the birds stayed a minimum of 5 cm from the object for 5 s. If no object selection was made, a result of 0 was recorded for each object.
- The tonic immobility test—the birds were placed on a “cradle” to induce their tonic immobility instinct. The time from the onset of immobility until the animal returned to its original state was measured.

After 6 weeks of the experiment, blood samples (1 mL/bird) were collected (by a qualified veterinarian) from 3 birds from each replication subgroup from the brachial vein using sterile and disposable syringes and placed in plastic tubes containing K3-EDTA. Whole blood samples were also smeared and fixed using a commercial Hemacolor^®^ stain kit (Sigma-Aldrich Co., Oakville, ON, Canada), according to the manufacturer’s recommended procedures. The preparations were observed under a DELTA^®^ optical microscope (Delta Optical, Nowe Osiny, Poland) at ×1000 magnification. The number of heterophils and lymphocytes was counted, according to the principles proposed by Lucas [20]. 

The samples of blood were centrifuged at 1000 RPM for 10 min. The plasma concentration levels of aspartate aminotransferase (AST), alanine aminotransferase (ALT) and lactate dehydrogenase (LDH) were assessed. The evaluation of the above-mentioned parameters was performed using a BS 130 Apparatus Mindray (Shenzhen Mindray Bio-medical Electronics Co., Shenzhen, China). 

The concentration of cortisol and corticosterone levels were also assessed using commercial analytical kits (BT Lab, Birmingham, UK). These concentrations were measured using a Synergy HTX microplate reader (BioTek Instruments, Inc., Winooski, VT, USA).

The SPSS 24.0 package [21] was used for the statistical processing of the obtained data. The normal distribution of the particular variables was verified using the Shapiro–Wilk test. In the case of normally distributed and measurable traits (i.e., the time of reaction, tonic immobility test data and blood parameters), the groups were compared using a one-way analysis of variance with Tukey’s multiple comparisons test. Traits not showing a normal distribution were analysed using the χ^2^ test of trait independence (i.e., the number of eggs, place of laying, number of fertile eggs and open-field test data).

## 3. Results

### 3.1. Nesting Behaviour and Fertility Rate

**Chart 1:**
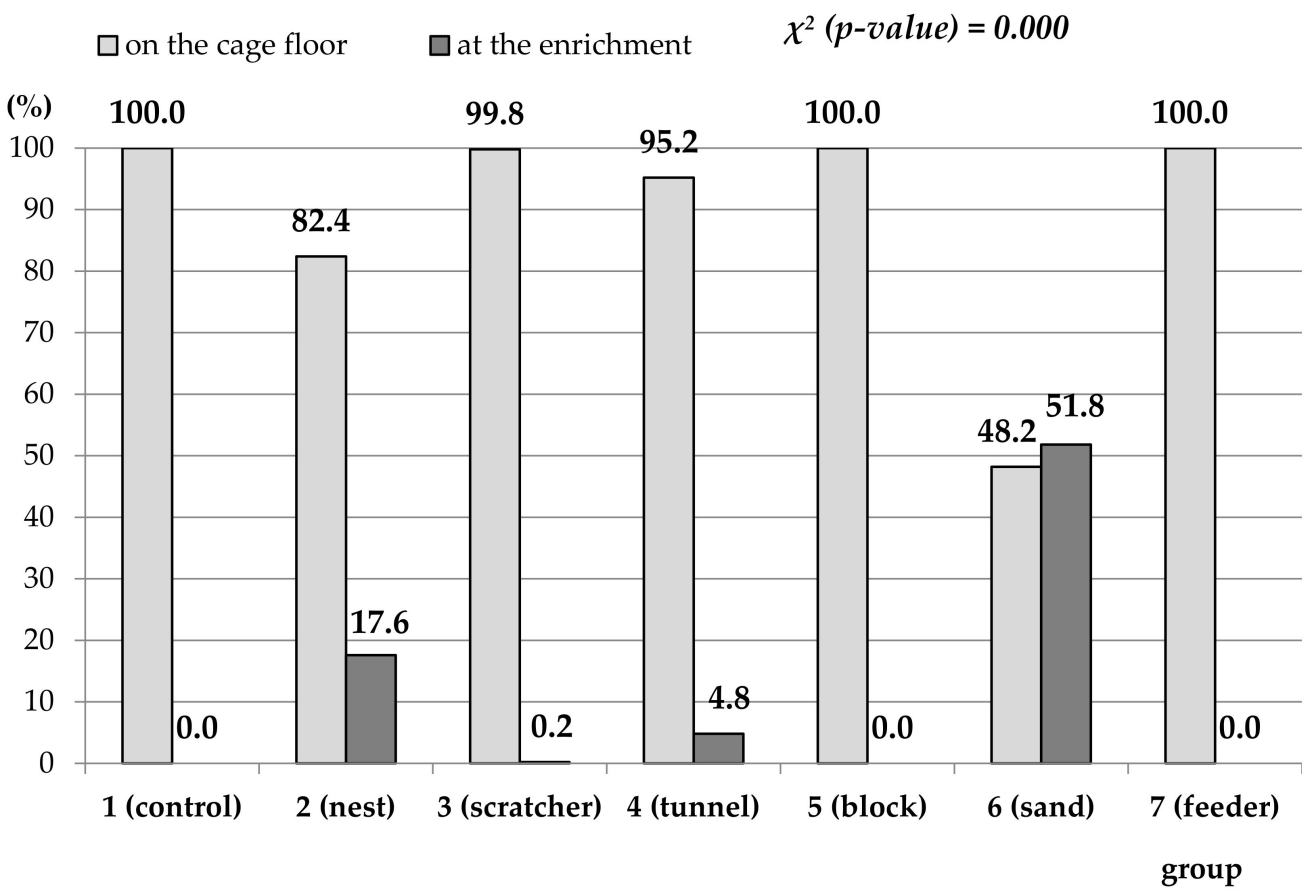
Egg-laying habitats of Japanese quails depending on the cage enrichment applied.

When the eggs were collected, the location of their laying was noted by distinguishing the area of the cage floor from the area of the enrichment (Chart 1). At the same time, it should be noted that, when the birds had the opportunity to lay eggs in the enrichment area, such a kind of behaviour was observed (groups 2, 3, 4, 6 and 7). In the case of group 7, no eggs were observed on the enrichment area. In group 3, egg-laying was observed on the enrichment area; however, the frequency was low. The most frequent egg-laying in the enrichment area was in group 6, equipped with a sand-bath box. 

**Chart 2:**
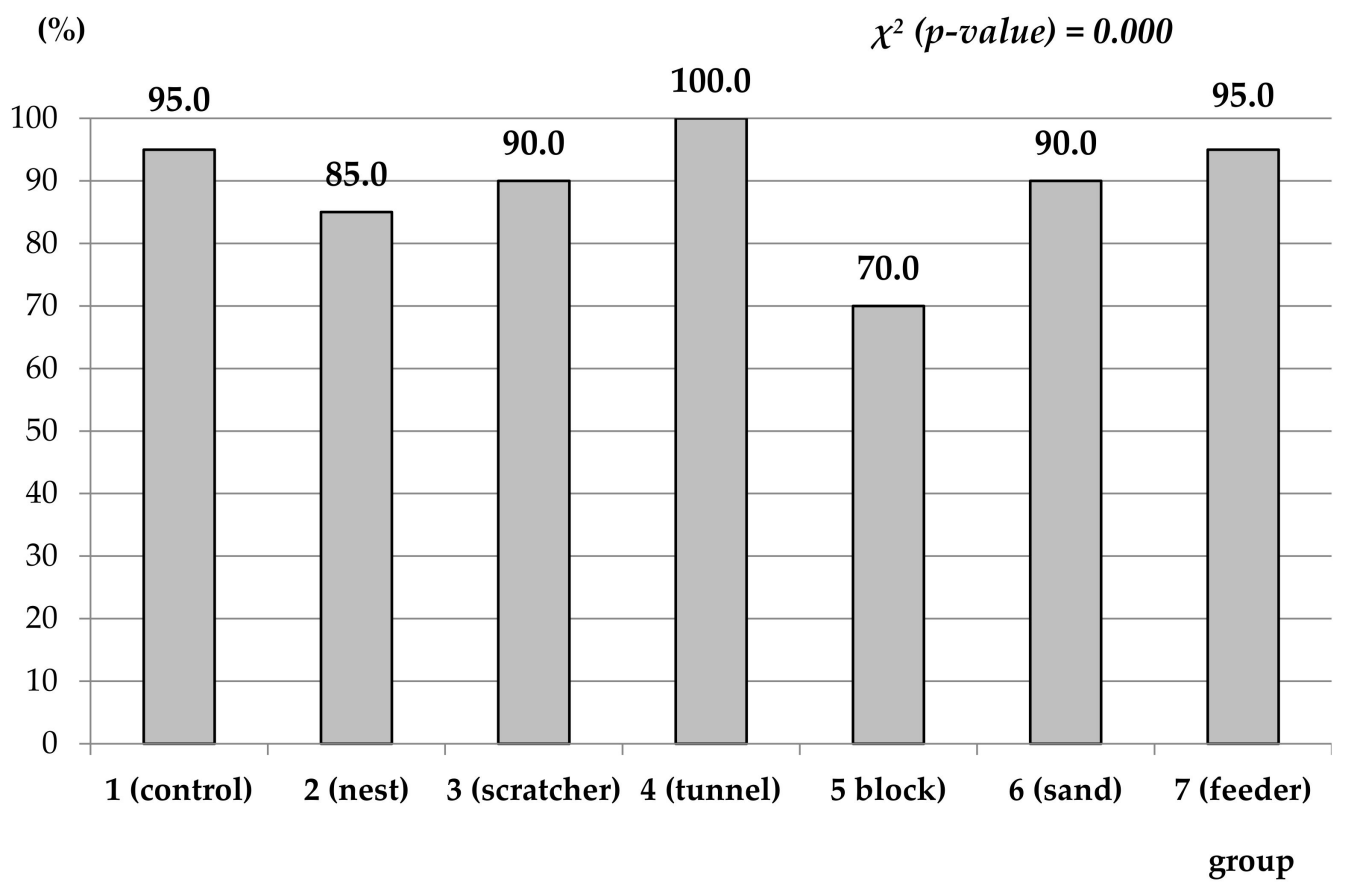
Results of the fertility analysis of Japanese quail eggs depending on the cage enrichment applied.

The visual assessment of egg fertility (after pre-incubation) showed that the percentage of fertile eggs significantly depended on the enrichment used. The best fertility rate characterised eggs obtained from group 4, which had a tunnel in their cage. In contrast, the lowest fertility rate was found in group 5, which had a limestone block at its disposal (Chart 2).

### 3.2. Behavioural Tests

Following a modified open-field test, the first reaction time, the time taken to choose the first object and the number of objects chosen during the test were analysed (Table 1). Significantly, the shortest first reaction time was found in groups 3 (scratcher), 4 (tunnel) and 6 (sand), while the longest time was found in group 5 (limestone blocks). Analysing the time that the birds required to choose the first object, it was observed that the shortest time was characteristic of birds from group 4 (tunnel), with the highest values for this feature among group 7 (feeder). Birds from groups 1, 2 and 5 did not differ significantly from one another, but they required much more time than the quails from group 4. In the case of the number of objects chosen during the test, group 7 (feeder) showed the significantly lowest value for this index, while the highest number of enrichments was chosen by birds from the cages equipped with a nest (2) and limestone blocks (5).

Analysing the second part of the open field test (Table 2), attention was paid to the following: the first object chosen, the object in which the most birds were interested and which they stayed at for the longest time, the occurrence of defecation, sexual behaviour, grooming behaviour, foraging, behavioural disturbance, empty sand-baths and environmental exploration. Of all the available objects, the scratcher was the most frequently chosen as the first object, approached by up to 35.7% of quails. It was the most preferred by birds in the control group, as well as those in the cages equipped with a nest (2), a scratcher (3) and blocks (5), while it was not the first choice among birds in groups 6 and 7. The second most popular object was the feeder, preferred by 32.1% of individuals. It was most frequently chosen by birds from groups 2, 3 and 6, while birds from the other groups were not interested in it at all, or only a few individuals were interested. The tunnel and the sand were chosen as the first object by 10.7% of birds, with the former being most frequently approached by birds from groups 4, 5 and 6, and the latter by birds from group 7, respectively. The block was least frequently chosen as the first object by 3.6% of birds from group 7 only. 

Throughout the test, the birds spent most of their time at three objects. Sand, chosen by 53.6% of the birds tested, was most frequently occupied by birds from group 6, whose cage was also equipped with a sand box. The rest of the groups studied showed similar results, i.e., 50%. The next most popular object was the scratcher, which attracted 28.6% of the birds studied, mostly from group 5. The other groups did not differ significantly. The last item was the feeder, chosen by 17.9% of birds, but it was most frequently approached by birds from groups 1, 2, 4, 6 and 7, while birds from groups 3 and 5 stayed at it for a relatively short time and in small numbers (Table 2). Defecation, which may be one of the stress measurements, occurred during the whole test in 17.9% of the birds tested, most frequently in group 1 (the control) and group 5 (block). It did not occur in 82.1% of individuals belonging to groups 2, 4, 6 and 7. In the arena, the sexual behaviour of the birds was also observed; it occurred most frequently (50%) in birds from group 5 (block). This behaviour was not observed in birds from groups 1 (the control), 3 (scratcher) and 6 (sand). In the other groups, it occurred much less frequently. Grooming behaviour was also present among the studied individuals, affecting as many as 89.3% of the birds, most frequently from group 1 (the control), 4 (tunnel), 5 (block) and 7 (feeder). The other groups either did not demonstrate this behaviour or engaged in it sporadically. The foraging instinct, the instinct to search for food while exploring the area, occurred in up to 75% of the quails tested, most frequently in groups 2 (nest) and 3 (scratcher).

Quail behaviour, further classified as behavioural disorder, was also observed. This occurred in 82.1% of the birds tested. These disturbances were most frequent in group 2 (nest), 4 (tunnel) and 7 (feeder), while in the remaining groups, they occurred only among single individuals. The most common behavioural disturbance observed was an absence of sandbathing, which occurred in 57.1% of birds, mostly in birds from group 2. The exploration of the environment was determined by evaluating the number of chosen items and the desire to move around the arena. It was often combined with the foraging instinct. Exploration alone occurred in 39.3% of the birds tested, with the highest prevalence in group 2 (nest), while it did not occur in animals from group 7 (feeder).

**Chart 3:**
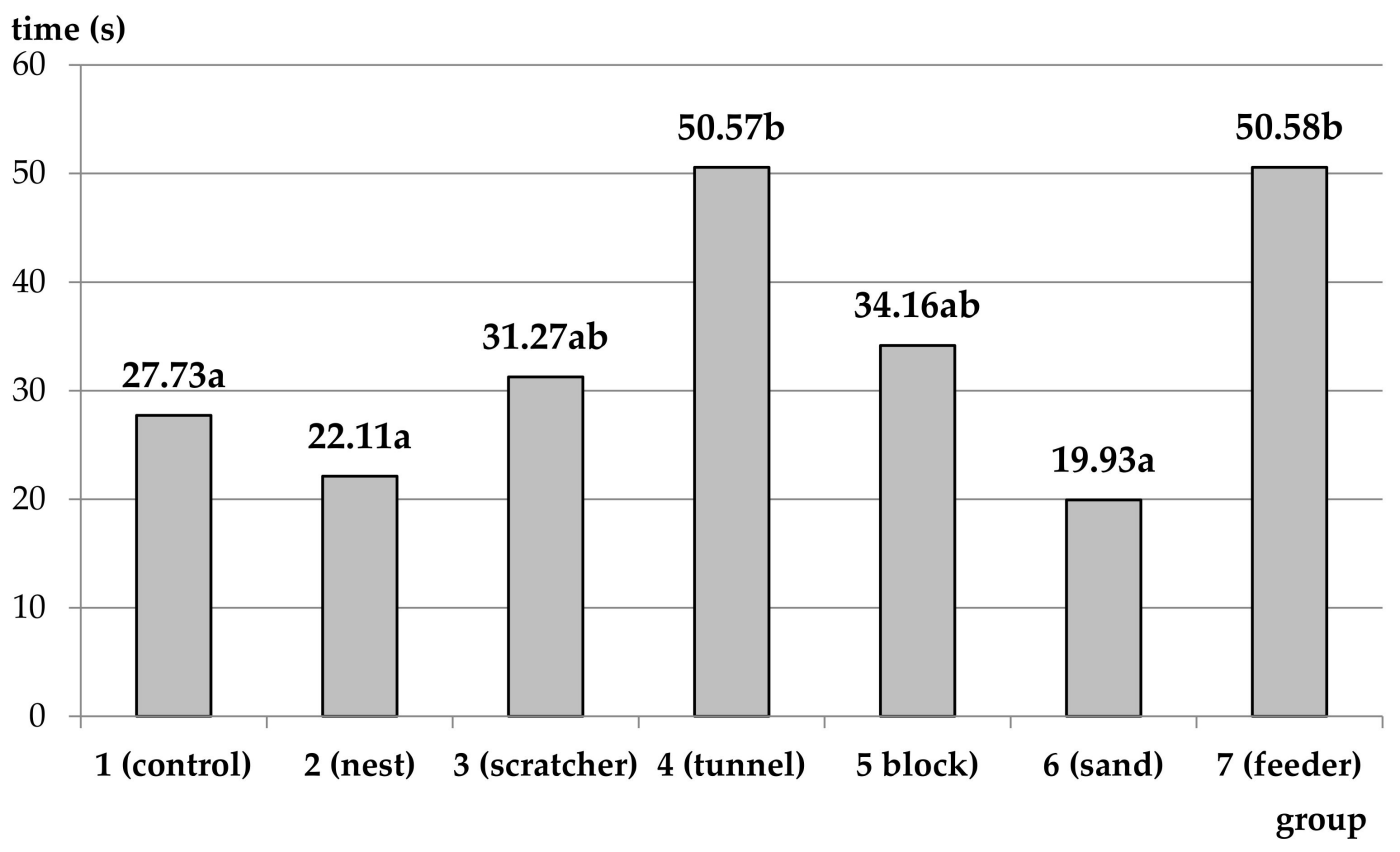
Results of the tonic immobility test of the Japanese quails depending on the applied cage enrichment. a, b—means (groups) differ significantly at *p* ≤ 0.05.

During the tonic immobility test, the time in seconds from the moment the bird was placed on the cradle and rendered immobile until it returned to its original position was recorded. The considerably longest time of tonic immobility (Chart 3) was observed in groups 4 (tunnel) and 7 (feeder), while the lowest value for this feature was recorded in the groups whose cages were equipped with a nest (2) and sand-bath box (6).

### 3.3. Blood Analyses

One of the most important stress-related indicators was the heterophil-to-lymphocyte ratio (H:L). The highest value for this trait was found in the control group (Table 3). No significant differences were found for the other groups included in the study. 

The level of cortisol concentration in the blood plasma (Table 3) showed significant variability depending on the type of enrichment with which the birds were maintained. The lowest level was identified in the blood of birds with access to sand, with the significantly highest level recorded for the control group. It is important to note that high levels of cortisol were also found in birds from the scratcher (3) and limestones block (5) groups. A slightly different relationship was observed for the corticosterone level. Birds maintained with access to sand (group 6) had the lowest levels of corticosterone concentration, with the highest values recorded for the control group. Access to sand (6), a scratcher (3) and a feeder (7) did not differentiate the groups in terms of the blood plasma corticosterone levels.

The analysis of the biochemical profile showed the highest ALT levels in the blood of birds maintained with access to a nest (2) and sand (6), with the lowest levels for the groups maintained in cages equipped with the scratcher and limestone blocks (5). As regards the aspartate aminotransferase, the highest values were recorded in the blood of quails from groups 4 (tunnel) and 7 (feeder). The experimental groups also differed in their lactate dehydrogenase (LDH) levels. The lowest level was recorded in groups 3, 5 and 7 (scratch, tunnel and feeder, respectively), with the highest value recorded in the group of birds maintained with access to limestone blocks.

## 4. Discussion

The choice of location for nesting plays an important role in laying hens. Studies carried out by Cooper and Appleby [22,23] have shown that, in order to lay eggs in a preferred place, hens are able to overcome certain obstacles, such as narrow gaps, while they most often choose darkened and sheltered spaces as nesting sites. In the case of Japanese quails, the data obtained indicate a similar relationship. The choice of these sites is probably related to an instinctive need motivated by the place of nesting (on the ground) among the wild ancestors of these birds [24].

One of the most important elements in successful poultry breeding is the maintenance of a high fertility rate and good-quality chicks. The fertility rate depends on many factors, starting with sexual behaviour [25], the age of the birds [26] and a properly balanced diet that is rich in antioxidants [27]. In our study, these factors were unified, so the observed differences exclusively resulted from the introduced enrichments or individual variability. Sexual behaviour in Japanese quails includes vocalisation, as well as fighting or aggression between males [28]. It is possible that the observations presented in this study are not only the results of changes introduced into the habitat of Japanese quails, but also of the individual characteristics of males in the experimental flocks.

It is possible to measure stress levels in animals by laboratory tests or by observation during behavioural tests. Depending on the design and purpose of a particular test, it is possible to verify curiosity or fear [29]. In our study, a modified open-field arena test was conducted to verify the birds’ reactions to objects that were new to them. Similar studies carried out on hens showed significant variation in birds’ responses depending on the breed or the utility type [9]. However, in our study, birds from the same parental flock were compared; hence, this factor was not taken into account. Nevertheless, the differences observed both in the time of the first reaction and in the choice of a specific object point to differences in locomotor and, indirectly, stress levels in birds from various groups. Hocking et al. [30] indicate that birds experiencing negative emotions do not express an interest in objects present in their environment. Similarly, Schütz et al. [31] emphasise that, in the case of strong emotional stimulation of a negative nature, animals are not able to explore a given environment for a long time. Both these arguments indicate that, irrespective of the experimental groups in our study, we were observing positive motivation; i.e., the birds made their choices out of curiosity, which may indirectly prove a relatively low level of stress.

A scratcher was most often chosen as the first object. It is probably that this behaviour was related to the object’s colour (yellow). Manser’s [32] research showed that birds perceive colours differently from humans and are more sensitive to colours closer to red. Similar conclusions were also reached by Taylor et al. [33], who analysed the influence of perch colour on the choice of nest site and the reaction rate in egg-laying hens. The birds responded faster and with a higher probability of success to perches in contrasting colours.

This observation seems to be obvious, considering the natural behavioural needs of gallinaceous birds, which are sandbathing birds. In the case of caged egg-laying hens, studies have shown that access to dustbathing had a positive effect on their welfare [34]. Similar observations have also been made in relation to Japanese quails. In a study by Miller and Monech [19], it was shown that, if the birds gain access to sand-baths, they spend up to one fifth of their daily activity in them. This behaviour may be related to both the natural need for grooming and to instinctive foraging [35]. These observations were, moreover, implemented by the Council Directive 1999/74/EC, laying down the minimum standards for the protection of laying hens [2]. While sandbathing is an instinctive behaviour, excessive grooming can be considered a relaxing or stress-reducing action [36]. This is also supported by the work of Kozak et al. [9], who showed that birds exhibiting more frequent grooming behaviour in the arena had significantly higher cortisol levels. Our observations indicate that grooming behaviour affected almost all birds, which could be a result of stress induced by placing the birds in an unknown environment. On the other hand, empty sand-baths were mostly related to birds maintained with a nest box, while birds with a sand container in their cage did not exhibit this behaviour. This may indicate that birds deprived of an element in which to hide (the nest) experienced significant stress, while those with constant access to a sand-bath were fully satisfied in this need.

Another test that indirectly enables the assessment of stress levels is the tonic immobility test. The behaviour observed during this test is based on the natural immobility behaviour of birds when they are attacked by a predator [37]. We observed that birds from groups 4 (tunnel) and 7 (feeder) remained motionless for the longest time. These observations may point to increased levels of stress in birds from these groups, as studies conducted on chicks showed a correlation between the length of tonic immobility and fearfulness [38].

One of the most classical physiological indicators of stress is the heterophil to lymphocyte ratio (H:L). Research indicates that the introduction of enrichment elements into the birds’ environment has a positive effect on this parameter [39]. More importantly, H:L is an excellent marker of long-term stress, since it provides a picture of the immune system response to chronic stress [40,41]. Among the physiological indicators of stress are some elements of the blood plasma biochemical profile, as well as cortisol or corticosterone levels. Biochemical indicators like AST, ALT and LDH are most often analysed in the contexts of heat or oxidative stress [42], but other authors show that the disruption of the basic biochemical blood profile can also be influenced by the rearing system [43] or the enrichment of the birds’ environment [44,45].

Plasma cortisol and corticosterone concentration levels are one of the more frequently addressed topics in studies on birds’ welfare. In Japanese quails selected for long- or short-term tonic immobility, a relationship was found between the bird genotype and the corticosteroid output [46]. It is known that keeping animals under chronic stress conditions affects not only their production parameters [47], but also the reproductive [48] and immune parameters [18]. One of the more effective ways to regulate the level of stress is to introduce elements of environmental enrichment for the animals. So far, positive effects of similar treatment methods have been reported, such as reducing the level of fear and the intensity of stress responses [49], reducing anxiety [19] or abnormal behaviour, and the frequency of stereotypies [50].

At the same time, the reduction of stress levels through the use of environmental enrichment elements also indirectly affects other aspects of husbandry. Nazar and Marin [18] showed an improvement in the resistance parameters. In terms of the maintenance of reproductive flocks, this problem is even more important, since it has been shown that the level of glucocorticosteroids in the blood of the mother translates to their deposition into the yolk, which translates into the behaviour of the obtained chicks [51], and it also reduces fertility and hatchability rates [52]. In this respect, our own research shows a reduction in both abnormal behaviour and the negative impacts of cage stress through the use of environmental enrichment.

## 5. Conclusions

Enrichments that enabled the manifestation of natural instincts considerably reduced the tonic immobility duration in Japanese quails, indicating their low stress levels. Similar observations also apply to cortisol and corticosterone levels. In both cases, the best results demonstrate the validity of using a sand box as part of the environmental enrichment. The use of the tunnel positively affected the fertility rate of the eggs, although in the case of sand, a scratcher or a feeder, the results also remained at a satisfactory level. In conclusion, it was confirmed that the enrichment of the rearing environment of Japanese quails (*Coturnix japonica*) has a positive effect on the behavioural welfare indices of birds, although the physiological responses depended on the type of object used.

## Figures and Tables

**Table 1:** Results of the open-field test analysis of the Japanese quails depending on the applied cage enrichment, part 1.

Group (Enrichment)	1 (Control)	2 (Nest)	3 (Scratcher)	4 (Tunnel)	5 (Blocks)	6 (Sand)	7 (Feeder)	SEM
Trait								
Time of 1st reaction (s)	27.25 ab	17.00 ab	7.00 a	5.50 a	40.50 b	7.00 a	21.00 ab	2.336
Time of 1st object choice (s)	174.00 cd	174.50 cd	123.25 bc	11.75 a	153.75 cd	57.25 ab	211.50 d	8.939
Number of objects chosen during the test	4.50 ab	4.75 b	3.75 ab	4.50 ab	4.75 b	4.00 ab	3.50 a	0.115

a,b,c,d—means (groups) differ significantly, at *p* ≤ 0.05; SEM- standard error of mean.

**Table 2:** Results of the open-field test analysis of the Japanese quails depending on the applied cage enrichment.

Group (Enrichment)		1 (Control)	2 (Nest)	3 (Scratcher)	4 (Tunnel)	5 (Block)	6 (Sand)	7 (Feeder)	Total	*χ^2^ Test (p-Value)*
Trait										
First object chosen (% of flocks)	nest	0.0	0.0	0.0	25.0	0.0	25.0	0.0	7.1	*0.000*
	scratcher	50.0	50.0	50.0	50.0	50.0	0.0	0.0	35.7	
	tunnel	0.0	0.0	0.0	25.0	25.0	25.0	0.0	10.7	
	block	0.0	0.0	0.0	0.0	0.0	0.0	25.0	3.6	
	sand	25.0	0.0	0.0	0.0	0.0	0.0	50.0	10.7	
	feeder	25.0	50.0	50.0	0.0	25.0	50.0	25.0	32.1	
object at which the most birds were located and for the longest time	scratcher	25.0	25.0	50.0	25.0	50.0	0.0	25.0	28.6	*0.075*
	sand	50.0	50.0	50.0	50.0	50.0	75.0	50.0	53.6	
	feeder	25.0	25.0	0.0	25.0	0.0	25.0	25.0	17.9	
defecation	yes	50.0	0.0	25.0	0.0	50.0	0.0	0.0	17.9	*0.000*
	no	50.0	100.0	75.0	100.0	50.0	100.0	100.0	82.1	
sexual behaviour	yes	0.0	25.0	0.0	25.0	50.0	0.0	25.0	17.9	*0.001*
	no	100.0	75.0	100.0	75.0	50.0	100.0	75.0	82.1	
grooming behaviour	yes	100.0	75.0	75.0	100.0	100.0	75.0	100.0	89.3	*0.006*
	no	0.0	25.0	25.0	0.0	0.0	25.0	0.0	10.7	
foraging	yes	75.0	100.0	100.0	75.0	75.0	25.0	75.0	75.0	*0.000*
	no	25.0	0.0	0.0	25.0	25.0	75.0	25.0	25.0	
behavioural disorders	yes	50.0	100.0	75.0	100.0	75.0	75.0	100.0	82.1	*0.001*
	no	50.0	0.0	25.0	0.0	25.0	25.0	0.0	17.9	
empty sand-baths	yes	25.0	100.0	75.0	75.0	50.0	0.0	75.0	57.1	*0.000*
	no	75.0	0.0	25.0	25.0	50.0	100.0	25.0	42.9	
environment exploration	yes	25.0	75.0	50.0	50.0	50.0	25.0	0.0	39.3	*0.001*
	no	75.0	25.0	50.0	50.0	50.0	75.0	100.0	60.7	

**Table 3:** Selected blood and serum parameters of the Japanese quails depending on the applied cage enrichment.

Group (Enrichment)	1 (Control)	2 (Nest)	3 (Scratcher)	4 (Tunnel)	5 (Block)	6 (Sand)	7 (Feeder)	SEM
Trait								
H:L	0.713 b	0.487 a	0.479 a	0.421 a	0.516 ab	0.338 a	0.502 a	0.019
Cortisol (ng/mL)	180.20 d	66.40 b	110.60 c	52.10 b	126.80 c	27.00 a	48.23 b	6.956
Corticosterone (ng/mL)	13.88 d	6.04 b	6.70 b	5.91 b	8.85 c	3.43 a	5.07 ab	0.455
ALT (U/L)	101.1 ab	257.7 b	0.200 a	85.00 ab	0.300 a	247.30 b	95.33 ab	42.11
AST (U/L)	270.90 ab	194.00 a	232.00 ab	305.00 b	260.50 ab	250.80 ab	293.30 b	18.79
LDH (U/L)	402.00 ab	528.20 ab	394.00 a	388.00 a	542.80 b	421.30 ab	379.50 a	40.46

a,b,c,d—means (groups) differ significantly at *p* ≤ 0.05; SEM- standard error of mean.

## Data Availability

The data presented in this study are available on request from the corresponding author.

## Supplementary Materials

The following supporting information can be downloaded at: https://www.mdpi.com/article/10.3390/ani12151963/s1, Table S1. The environmental enrichments applied to Japanese quail cages.
