# Supplementary material for: Environmental Enrichment as Part of the Improvement of the Welfare of Japanese Quails
Source: Animals (Basel). 2022 Aug 2;12(15):1963. doi: 10.3390/ani12151963 (PMC9367415; doi:10.3390/ani12151963)
Supplement: Supplementary file 1 [file animals-12-01963-s001.zip › animals-1796374-supplementary.pdf]

Table S1. The environmental enrichments applied to Japanese quails cages.

Environmental enrichments	Enrichment's description	Schematic visualisation of the proposed enrichment
Nesting box	Plastic container, non-transparent, sized approx. 25x25x15cm, with cut-outs (2) of 7 cm diameter, placed on the floor of the cage	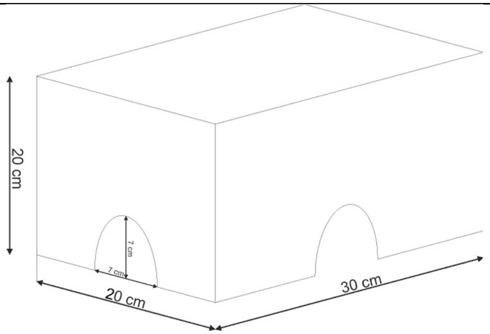
Scratching area	Plastic pine needle mat (artificial grass type), sized 15x15 cm, attached to the floor of the cage	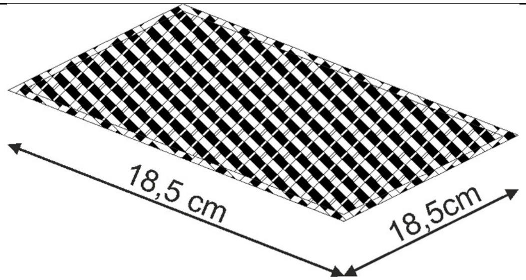
Tunnel	Plastic corrugated pipe, flexible, 60 cm long and approx. 15 cm in diameter, laid on the bottom of the cage so that there is at least one joint.	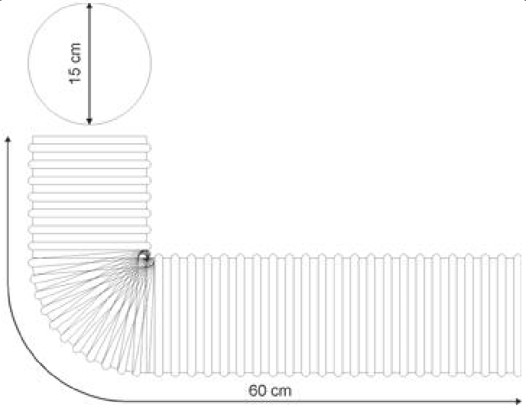

Limestone blocks	Limestone cubes are attached to the cage walls so that the bottom edge of the cube is 7 cm above the bottom of the cage.	
Sand bathing container	Transparent plastic container, dimensions approx. 25x25x25cm, with lid. Circular cut-out at the height of 5 cm from the base, diameter 7 cm; filled up to the height of 2.5 cm from the base with loose material (autoclaved sand)	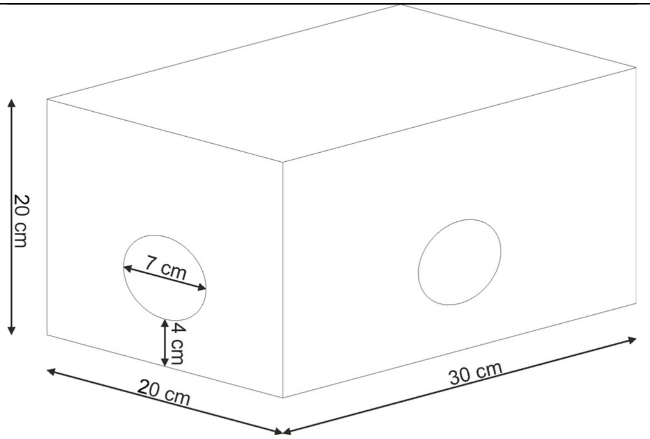
A feeder with variable diameter holes	Plastic container, dimensions approx. 15x21x3 cm, non-transparent, with lid. Variable diameter holes (0.2; 0.3; 0.5; 0.7 cm) in the lid.	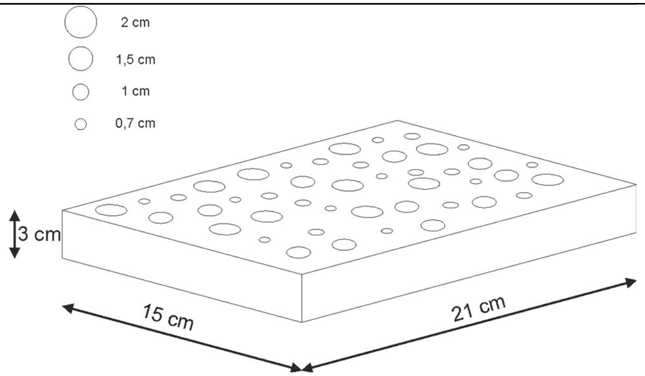
